# Effect of a Multimedia-Assisted Informed Consent Procedure on the Information Gain of Patients Undergoing Mastectomy and Implant-Based Reconstruction

**DOI:** 10.1007/s00266-025-05493-5

**Published:** 2025-12-14

**Authors:** Francesca De Lorenzi, Riccardo Carbonaro, Chiara Marzorati, Eleonora Pagan, Sergio Miranda, Vincenzo Bagnardi, Claudia Sangalli, Mara Negri, Gabriella Pravettoni, Paolo Veronesi

**Affiliations:** 1 Division of Plastic and Reconstructive Surgery, IEO, European Institute of Oncology, IRCCS, Milan, Italy; 2 Università degli Studi di Milano, Via Festa del Perdono 7, 20122 Milan, Italy; 3 Applied Research Division for Cognitive and Psychological Science, European Institute of Oncology, IRCCS, Milan, Italy; 4 Department of Statistics and Quantitative Methods, University of Milano-Bicocca, Milan, Italy; 5 Clinical Trial Office, European Institute of Oncology IRCCS, Milan, Italy; 6 Division of Breast Surgery, IEO, European Institute of Oncology, IRCCS, Milan, Italy; 7 Plastic Surgery Department, I.R.C.C.S. Istituto Ortopedico Galeazzi, Via Cristina Belgioioso 173, 20157 Milan, Italy

**Keywords:** Breast, Informed consent, Multimedia, Information

## Abstract

**Introduction:**

Implant-based reconstruction is the most frequent procedure after mastectomy. Effective preoperative counseling and a thorough informed consent process are crucial for informing patients about oncologic surgery, reconstruction options, and expected cosmetic outcomes. Recent studies indicate that a multimedia video-assisted informed consent procedure may enhance patient information retention compared to traditional methods. This study aims to compare the conventional informed consent process, supplemented with a written informational brochure, to a multimedia video-assisted approach.

**Materials and Methods:**

From January to June 2024, 265 consecutive breast cancer patients scheduled for mastectomy and implant-based reconstruction at the European Institute of Oncology in Milan, Italy, were enrolled in this controlled randomized prospective study. Of these, 200 patients completed evaluation questionnaires assessing information retention and anxiety. A six-minute video featuring simple schematic illustrations and automated text-to-speech narration in Italian was developed to enhance understanding of the risks, benefits, and alternatives of the surgical treatment. Patients were randomly assigned to two groups: Group A received the video presentation along with evaluation questionnaires via email, while Group B received only the questionnaires.

**Results:**

Patients in the multimedia video-assisted group demonstrated significantly higher overall comprehension compared to those in the control group. Although scores from the Spielberger State/Trait Anxiety Inventory (STAI) and the Decisional Conflict Scale (DCS) indicated greater anxiety and decisional conflict in the standard group, these differences were not statistically significant.

**Conclusions:**

The multimedia video-assisted informed consent process is an effective tool for enhancing patient knowledge and awareness regarding implant-based breast reconstruction. This method improves information uptake and retention, suggesting its superiority over traditional communication techniques in preoperative counseling. These findings support the integration of multimedia resources in patient education to facilitate better-informed decision-making.

**Level of Evidence II:**

This journal requires that authors assign a level of evidence to each article. For a full description of these Evidence-Based Medicine ratings, please refer to the Table of Contents or the online Instructions to Authors www.springer.com/00266.

**Supplementary Information:**

The online version contains supplementary material available at 10.1007/s00266-025-05493-5.

## Introduction

Implant-based reconstruction is the most frequently performed procedure following mastectomy, preserving body integrity and femininity, and accounting for approximately 81% of post-mastectomy reconstructions in the United States [1]. Breast reconstruction is considered a right for women in many countries, with documented positive psychological effects [2]. It forms an integral part of breast cancer treatment and care and is predominantly performed immediately after mastectomy [3]. Advances in screening programs, modern mammographic imaging, and increased awareness among women have contributed to earlier cancer diagnosis, enabling conservative mastectomies, and immediate reconstruction [4].

Breast reconstruction is tailored to each patient, considering anatomy, comorbidities, cancer stage, oncologic guidelines, and patient preferences. Given the high expectations for cosmetic outcomes, particularly in prophylactic surgery, preoperative counseling is essential. It provides information on oncologic surgery, reconstruction options, and expected results, enabling patients to actively participate in clinical decisions, addressing doubts, and reducing surgery-related anxiety [5–7].

At our Institute, after the initial consultation with the breast surgeon, preoperative counseling involves medical and instrumental evaluations to determine the appropriate cancer surgery. Counseling with a plastic surgeon is mandatory to discuss reconstructive options and devise an individualized reconstruction plan. The informed consent process includes explanations of surgical approaches, their benefits, alternatives, risks, implant options (temporary or definitive), and postoperative scenarios [8]. This is supported by informational brochures, though these documents often suffer from poor readability and low information retention rates [9, 10].

Recent research indicates low health literacy levels across Europe, with 33–50% of individuals struggling to understand basic health information [11, 12]. A systematic review and meta-analysis revealed that visual-based interventions, such as multimedia videos, are more effective than written materials in improving comprehension of health-related information [13], This approach has been successfully applied in cataract surgery [14, 15], trauma surgery [16], urologic procedures [17], gynecologic surgery [18], and other surgical fields [19].

Building on this evidence, the current study seeks to evaluate whether a multimedia-assisted video tutorial improves information retention in patients undergoing mastectomy followed by immediate implant-based breast reconstruction. The study compares the conventional informed consent process, supported by a written brochure, with a multimedia-enhanced procedure incorporating a specific informational video and the same brochure. Outcomes will be measured in terms of information retention and knowledge regarding implant-based reconstruction, with secondary objectives assessing the impact on patient anxiety and decision-making quality.

## Materials and Methods

### Study Design and Population

This prospective randomized study was conducted between January and June 2024 at the European Institute of Oncology in Milan, Italy. A total of 265 consecutive patients were identified as candidates for mastectomy and subsequent implant-based reconstruction. These reconstructions included definitive implants, temporary prostheses, or expanders. The study population comprised women undergoing surgery for breast cancer, as well as those identified as high-risk individuals who elected to undergo prophylactic, risk-reducing mastectomies.

Eligible patients included those undergoing unilateral or bilateral mastectomies. In cases of unilateral mastectomy, individuals were included regardless of whether they required additional contralateral mammaplasty for symmetry of the healthy breast. However, women who underwent mastectomy with planned autologous reconstruction were excluded. Similarly, patients with a history of unilateral mastectomy and implant reconstruction who subsequently required contralateral mastectomy were not eligible for participation.

### Preoperative Consultation and Patient Counseling

All participants underwent a mandatory pre-admission consultation with a plastic surgeon as part of their comprehensive preoperative evaluation. During this session, patients were evaluated to determine the most suitable reconstruction technique based on multiple factors, including their anatomy, cancer stage, comorbidities, and individual preferences. Reconstruction options discussed included expander-based reconstruction, direct-to-implant procedures, and various approaches such as submuscular, dual-plane, or prepectoral reconstructions.

The consultation emphasized the benefits and limitations of each technique, potential aesthetic outcomes, alternative surgical approaches, and possible postoperative complications. Patients were encouraged to ask questions to address any uncertainties or concerns. To support their understanding, a hospital-approved informational brochure summarizing all reconstructive options and associated details was provided. This document was reviewed during the consultation to ensure clarity and alignment with patient expectations.

The preoperative counseling process was conducted by a multidisciplinary breast team consisting of several plastic surgeons who collaboratively contributed to patient evaluations and education. This collaborative approach ensured a comprehensive discussion tailored to each patient’s specific medical and psychological needs.

### Informed Consent and Study Enrollment

Patients meeting eligibility criteria were informed about the study by their consulting plastic surgeon. The study’s objectives, procedures, and potential benefits were explained in detail, and patients who agreed to participate provided specific informed consent. At the time of enrollment, participants also completed a baseline questionnaire collecting sociodemographic data.

Exclusion criteria for study participation included patients unable to see, hear, or comprehend the multimedia video presentation or those unavailable for electronic correspondence, which was integral to the study’s evaluation process.

### Randomization Procedure

Participants were prospectively randomized into two study groups:

- Group A received a multimedia video presentation on implant-based reconstruction delivered via email. In addition, they were asked to complete evaluation questionnaires designed to assess their retention of information and levels of anxiety.
- Group B received only the evaluation questionnaires and did not view the multimedia video presentation.

The questionnaire was not administered right after the consultation with the plastic surgeon for the purpose of testing information retention at least after 24h, which is the minimum time passing normally from preop consultation with the Plastic Surgeon to surgery. Moreover, randomization was never performed prior to the consultation with the Plastic Surgeon to ensure objectivity. In fact, the physician conducting the face-to-face informed consent procedure was blinded to the randomization assignment. This approach prevented bias in the interaction between the healthcare provider and the patient during the standard informed consent process.

All questionnaires were sent to the patient via email, were completed at home with clear instructions, and were automatically uploaded to our server where the file was easily accessible facilitating not only the statistical analysis but also avoiding any loss of data and or lack of privacy possibly associated to printed paper questionnaires.

The randomization list was prepared by the study statistician using the R package “blockrand.” Block sizes varied from 2 to 8 to maintain randomization balance across the groups [20].

### Patient Exclusions and Final Sample Size

Out of the initial 265 patients enrolled in the study, only 200 completed the evaluation questionnaires in their entirety and were included in the final analysis. Reasons for patient withdrawal included:

- 56 patients who were unable to complete the questionnaires before hospital admission.
- 5 patients who decided to undergo autologous reconstruction after enrollment.
- 3 patients whose surgical plans were modified before admission.
- 1 patient who declined immediate reconstruction.

**Fig. 1:**
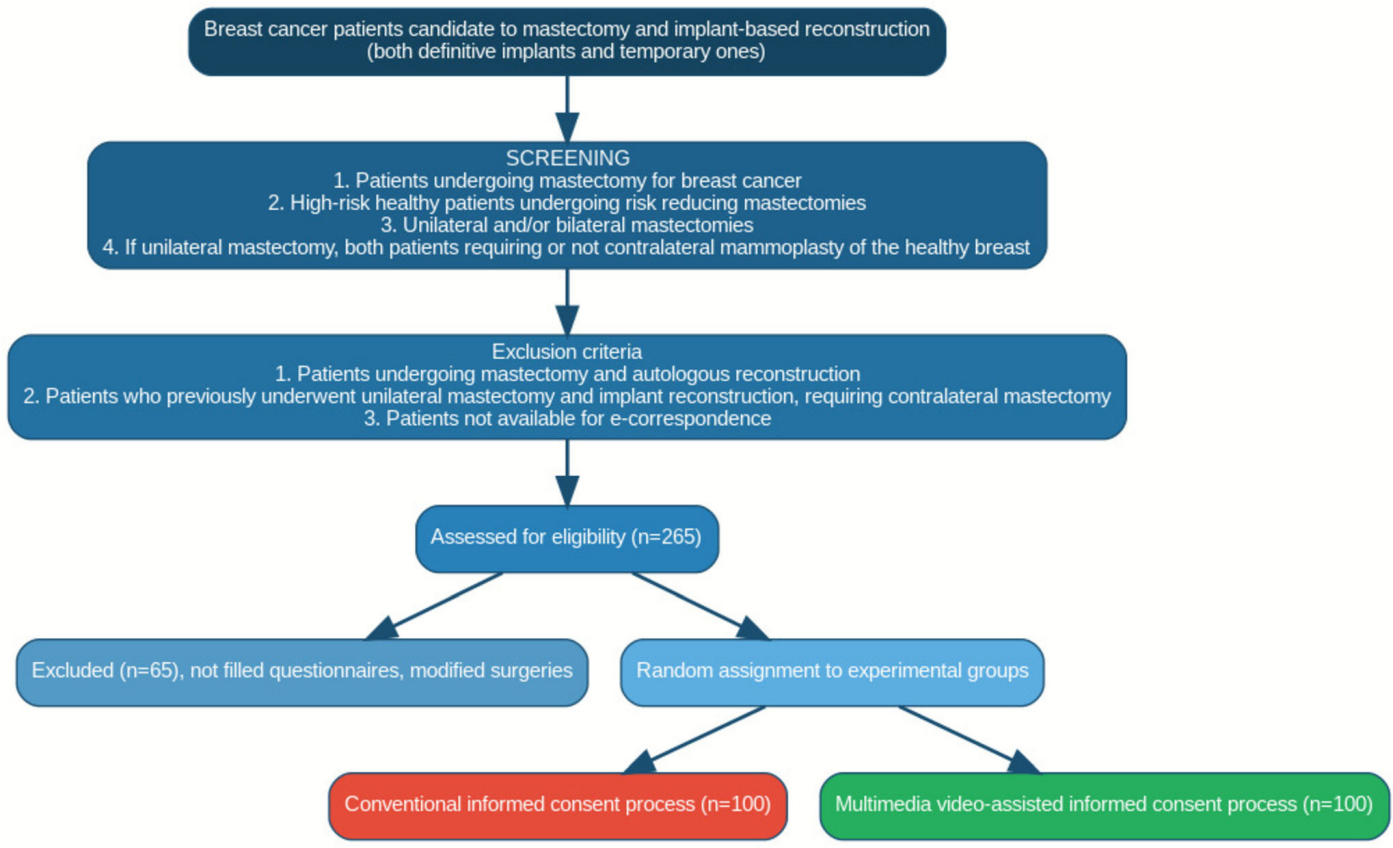
Flowchart of patient enrollment and randomization process

The final sample size ensured robust statistical analysis and sufficient power to detect meaningful differences between the study groups (see Fig. 1, Study Scheme Diagram).

### Multimedia Video Tool

A 6-minute multimedia video was developed specifically for this study. The video featured simple schematic drawings created by the first author (FDL) and automated text-to-speech narration in the patients’ native language (Italian). Key topics included an overview of the surgical procedures, the risks and benefits of implant-based reconstruction, and available alternatives.

Animations and diagrams were incorporated to visually explain complex concepts, such as implant placement, the distinction between skin-sparing and nipple-areola-sparing mastectomies, and the mechanics of postoperative recovery. To enhance understanding, key points were reinforced using on-screen instructional text displayed alongside the narration.

Patients had the flexibility to navigate through the video, pausing, and revisiting sections as needed, ensuring they could review the material multiple times to solidify their comprehension. The video’s content was aligned with the information in the brochure distributed during the initial consultation (Supplemental Video).

### Outcome Measures

To evaluate the effectiveness of the multimedia-assisted informed consent process, patients completed three questionnaires.

1. *Spielberger State/Trait Anxiety Inventory (STAI)* [21]: This widely validated tool measures anxiety levels in two dimensions: trait anxiety (general proneness to anxiety) and state anxiety (current anxiety level). The questionnaire comprises two subscales (STAI-Y1 and STAI-Y2), each containing 20 items rated on a 4-point Likert scale. Higher scores indicate greater anxiety. STAI was administered after the multimedia video or standard brochure was provided.
2. *Decisional Conflict Scale (DCS)* [22]: This scale assesses patient uncertainty in decision-making, contributing factors, and perceptions of effective decision-making. It uses a 5-point Likert scale ranging from “strongly agree” to “strongly disagree,” with higher scores indicating greater decisional conflict.
3. *Reconstructive Breast Surgery Comprehension Questionnaire*: This custom questionnaire was designed to assess patients’ understanding of reconstruction procedures and surgical complications. It included 5 multiple-choice questions addressing surgical incisions, implant placement, and potential complications, with higher scores reflecting better comprehension. Additionally, 6 Likert-scale questions assessed self-perceived comprehension, ranging from “not clear at all” to “very clear.”

### Sample Size and Statistical Considerations

As our study has multiple primary outcomes, we calculated the sample size considering the effect size as the main measure of interest. Referring to the thresholds commonly used in clinical and psychological research: an intervention effect is considered small if the effect size is 0.2, moderate if it is 0.5, and large if it is equal to or greater than 0.8 [23]. The primary outcome of the study was the effect size of the multimedia intervention on information retention and anxiety. An effect size of 0.5 or greater was hypothesized as moderate. To detect this effect size with 80% power and a Type I error of 1%, a minimum of 92 patients per group was required. Anticipating a dropout rate of less than 10%, the study aimed to recruit 100 patients per group.

Statistical analyses included Chi-square tests for categorical data, Cochran–Armitage trend tests for Likert-scale questions, and T-tests for continuous variables such as STAI and DCS scores. Analyses were performed using SAS software v9.4.

### Data Collection and Security

Patient data were collected via email and stored in a secure REDCap® database. This web-based platform supports data entry, storage, and export while ensuring compliance with security and audit standards. Further information about REDCap’s security features is available at http://www.projectredcap.org/.

## Results

Patients’ characteristics were well balanced between the two groups (Table 1). No significant differences were observed in age, employment status, education level, marital status, or motherhood. The overall mean age at enrollment was 48.7 years.

**Table 1:** Patients demographic characteristics

	Multimedia group (*N* = 100)		Control group (*N* = 100)	
	N	%	N	%
Age, median (IQR)	48.1 (39.7–56.2)		49.0 (43.6–53.9)	
Education level				
None	1	1.0	0	–
Primary school	1	1.0	1	1.0
Secondary school	23	23.0	10	10.0
High school	35	35.0	55	55.0
University education	40	40.0	34	34.0
Employed				
No	26	26.0	20	20.0
Yes	70	70.0	78	78.0
Missing	4	4.0	2	2.0
Type of work activity				
Laborer	4	4.0	5	5.0
Employee	40	40.0	49	49.0
Entrepreneur	6	6.0	8	8.0
Freelancer	8	8.0	11	11.0
Self-employed worker	6	6.0	2	2.0
Other	6	6.0	3	3.0
Missing	30	30.0	22	22.0
Motherhood				
Missing	0	–	2	2.0
No	22	22.0	25	25.0
Yes	78	78.0	73	73.0
N of children				
1 child	22	28.2	23	31.5
2 children	42	53.8	41	56.2
3+ children	12	15.4	8	11.0
Missing	2	2.6	1	1.4
Dependent children				
No	16	20.5	5	6.8
Yes	56	71.8	63	86.3
Missing	6	7.7	5	6.8
Living with husband or partner				
No	23	23.0	20	20.0
Yes	71	71.0	74	74.0
Missing	6	6.0	6	6.0

**Fig. 2:**
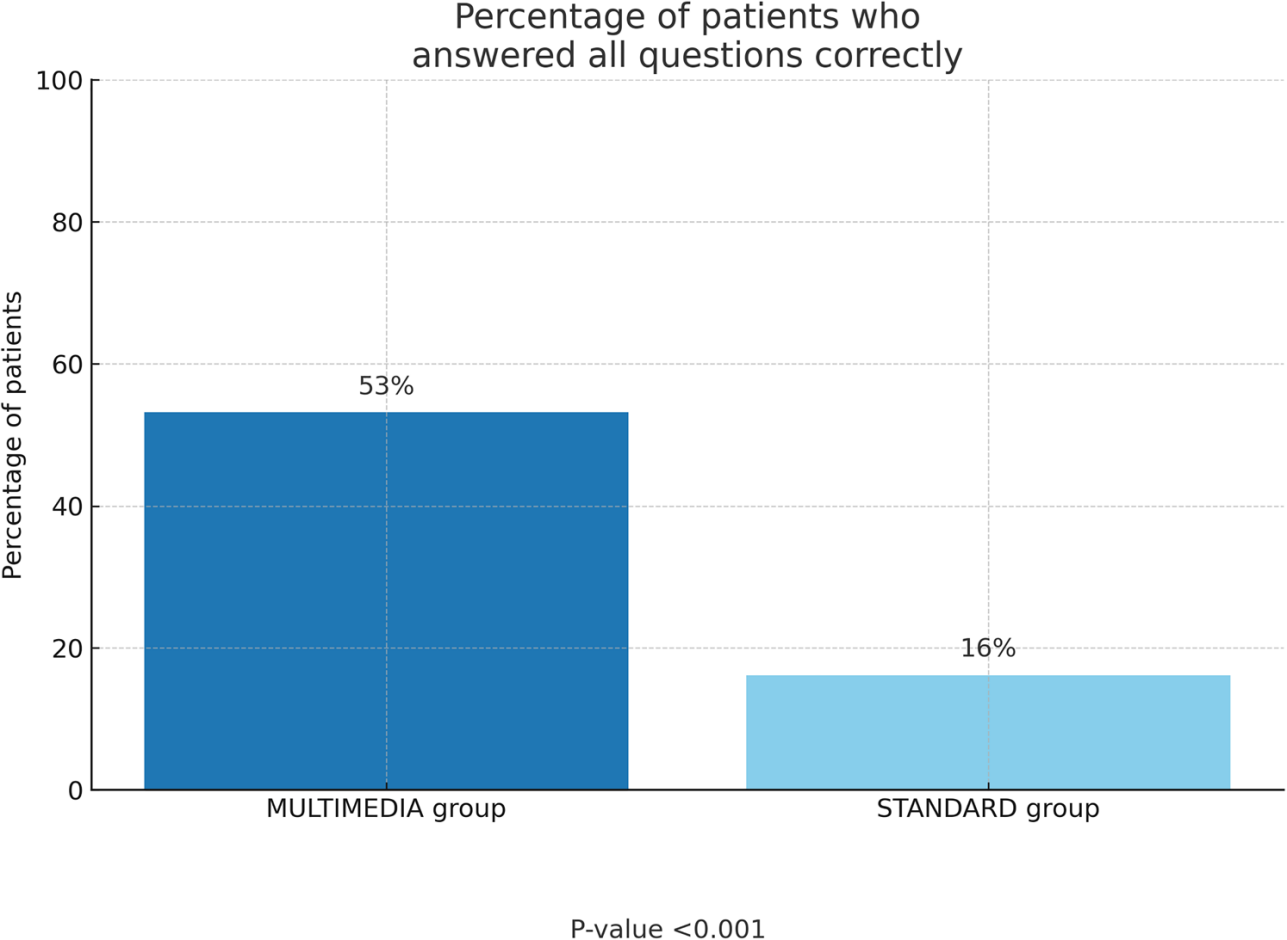
Percentage of patients who answered all questions correctly in the two groups

In the multiple-choice questions requiring patients to select the correct answer, the multimedia-assisted group demonstrated significantly better comprehension and retention of information concerning mastectomy skin incisions, types of mastectomy, and implant placement. Specifically, 53% of patients in the multimedia group answered all questions correctly, compared to only 16% in the control group (*p*-value < 0.001; Fig. 2).

Notably, significant differences in retention were observed for the statements: “the surgical access options for mastectomy and breast implant placement are radial, periareolar, or at the inframammary fold” and “the prepectoral breast reconstruction technique is less invasive and leads to greater advantages in postoperative recovery.”

Regarding the six Likert-type questions assessing self-perception of heterologous reconstruction options, temporary versus definitive implants, implant rupture, and postoperative surveillance, at least 60% of patients in both groups rated all questions as “very clear.” Notably, patients in the multimedia video-assisted group demonstrated significantly higher overall comprehension compared to the control group. Specifically, there was a significant difference in participants’ self-assessed understanding of “risk of implant rupture and need for post-op surveillance with annual ultrasound examination.” (Table 2).

**Table 2:** Reconstructive breast surgery comprehension questionnaire

	Multimedia group (*N* = 100)		Control group (*N* = 100)		*P*-value
	N	%	N	%	
Demolitive surgery (or mastectomy) consists of the removal of:					0.11^*^
The entire mammary gland and the breast tissue underlying the nipple	85	85.0	78	78.0	
The mammary gland alone; the nipple is always preserved	8	8.0	4	4.0	
Part of the mammary gland and the nipple	0	–	3	3.0	
All of the above	5	5.0	15	15.0	
Missing	2	2.0	0	–	
The surgical access modalities for breast implant placement are:					<0.001^*^
Radial	11	11.0	18	18.0	
Periareolar	7	7.0	4	4.0	
Inframammary fold	11	11.0	28	28.0	
All of the above	66	66.0	34	34.0	
Missing	5	5.0	16	16.0	
Breast reconstruction with implants include:					0.03^*^
Always an immediate reconstruction with definitive implants	1	1.0	3	3.0	
Always a reconstruction with temporary expander followed by a second stage in which expander are substituted with implants	0	–	4	4.0	
The placement of a permanent or temporary prosthesis according to the patient’s characteristics	97	97.0	92	92.0	
Missing	2	2.0	1	1.0	
We ask you to indicate your level of understanding regarding the following statements:					
It is not always possible to use the definitive prosthesis. Depending on anatomical features and surgical needs, sometimes the expander is used					0.09^ǂ^
Slightly/not at all clear	1	1.0	2	2.0	
Fairly clear	24	24.0	34	34.0	
Very clear	75	75.0	64	64.0	
The expander is always replaced with a permanent prosthesis					0.11^ǂ^
Slightly/not at all clear	3	3.0	6	6.0	
Fairly clear	24	24.0	31	31.0	
Very clear	73	73.0	63	63.0	
In some cases, a few years after implantation, the prosthesis may go through wear and tear processes, may break, and therefore may need to be replaced					0.11^ǂ^
Slightly/not at all clear	3	3.0	6	6.0	
Fairly clear	24	24.0	31	31.0	
Very clear	73	73.0	63	63.0	
Most patients find out about the rupture of the implant only incidentally through ultrasound follow-up					0.001^ǂ^
Slightly/not at all clear	12	12.0	24	24.0	
Fairly clear	28	28.0	38	38.0	
Very clear	60	60.0	37	37.0	
Missing	0	–	1	1.0	
The type of reconstruction is always individualized and depends on both the characteristics of the disease and the physical and anatomical features of the patient					0.11^ǂ^
Slightly/not at all clear	0	–	1	1.0	
Fairly clear	20	20.0	28	28.0	
Very clear	80	80.0	71	71.0	
There are two options for positioning of the definitive prosthesis (behind or in front of the pectoral muscle), and the choice is based on the patient’s characteristics and tissue quality					0.20^ǂ^
Slightly/not at all clear	5	5.0	10	10.0	
Fairly clear	27	27.0	28	28.0	
Very clear	68	68.0	61	61.0	
Missing	0	–	1	1.0	
The prepectoral technique is less invasive and leads to greater advantages in postoperative recovery					0.002^*^
True	81	81.0	63	63.0	
False	12	12.0	29	29.0	
Missing	7	7.0	8	8.0	
After mastectomy and reconstruction with prosthesis (both permanent and temporary)					0.16^*^
There are always some surgical complications	0	–	0	–	
There may be surgical complications	98	98.0	98	98.0	
There are no complications	0	–	2	2.0	
Missing	2	2.0	0	–	
Of the following listed surgical complications, is any of them unclear? (more than one answer allowed)					
Infection	5	5.0	14	14.0	0.03
Bleeding	12	12.0	11	11.0	0.82
Skin necrosis	28	28.0	26	26.0	0.75
Capsular contracture	29	29.0	47	47.0	0.009
In answering the questions above, did you need to look again at the informative material provided to you?					0.02
Yes, just once more	22	22.0	25	25.0	
Yes, several times I went to verify the information in the informative material	8	8.0	18	18.0	
No	62	62.0	57	57.0	
Other	6	6.0	0	–	
Missing	2	2.0	0	–	

^*^P-value for the comparison of response percentages for the correct answer toward all other options

^ǂ^*P*-value for the Cochrane-Armitage test for the trend

To answer the questionnaires, 62% of subjects in the multimedia group watched the video only once, 22% viewed it twice, and only 16% watched it more than twice. In the control group, 57% of the subjects read the written informational booklet only once, 25% read it twice and only 18% read the written informational material more than twice.

With regard to the Spielberger State/Trait Anxiety Inventory (STAI) questionnaire and the Decisional Conflict Scale, despite higher scores, suggesting greater anxiety and higher decisional conflict, were recorded in the standard group compared to the multimedia group, the difference between the two groups was not statistically significant. Mean score STAI-Y1 (anxiety state) was 2.1 (SD = 0.2) in multimedia group compared to 2.2 (SD = 0.2) in control group (*p*-value = 0.69) and DCS total score 19.0 (SD = 11.4) in multimedia group and 21.4 (SD = 15.5) in control group (*p*-value = 0.21) (Table 3).

**Table 3:** STAI-Y and decisional conflict scale (DCS)

	Multimedia group (*N* = 100)		Control group (*N* = 100)		*P*-value
	Mean	SD	Mean	SD	
STAI-Y1 anxiety state	2.1	0.2	2.2	0.2	0.69
STAI-Y2 anxiety trait	2.2	0.2	2.2	0.2	0.93
DCS total score	19.0	11.4	21.4	15.5	0.21
DCS uncertainty subscore	20.7	18.1	23.5	21.9	0.32
DCS informed subscore	15.8	19.3	19.0	19.9	0.24
DCS values clarity subscore	14.5	16.2	18.5	20.2	0.12
DCS support subscore	11.0	12.6	11.6	16.9	0.78
DCS effective decision subscore	29.8	9.3	31.3	13.0	0.33

## Discussion

Our study demonstrates that a multimedia video-assisted informed consent procedure enhances information retention among women undergoing mastectomy and implant-based reconstruction. To our knowledge, this is the first study using an audiovisual information tool specifically for this patient subgroup.

Several factors may contribute to the positive impact on comprehension. First, the video incorporates schematic drawings and animations that simplify the surgical procedure, making it easier to understand, even for patients with lower educational backgrounds. Second, it showcases various types of breast implants—round and anatomical, smooth, and textured—helping patients visualize the options available. Third, the video delivers comprehensive information about the entire surgical process, including postoperative complications and surveillance, which may improve patients’ self-perception of their knowledge.

Moreover, the video allows patients to rewind and rewatch segments as needed, and we believe that this opportunity for repeated viewing may boost their confidence regarding the reconstructive procedure. However, our findings indicate that 62% of subjects in the multimedia group watched the video only once, 22% viewed it twice, and only 16% watched it more than twice, suggesting that the content may have been sufficiently clear.

It is important to note that while the video serves as an illustrative and generalized tool, breast reconstruction increasingly requires a patient-tailored approach. Although the video standardizes information about breast reconstruction, face-to-face discussions with the plastic surgeon remain crucial for addressing each patient’s individualized reconstructive plan. In our study, the video was presented after the physician consultation, ensuring it complemented rather than replaced direct verbal communication. We believe that standardizing information through multimedia tools could be particularly beneficial for multicentric studies, helping to minimize site-specific variations.

We strongly support the use of this video-assisted tool to improve the information transfer from the surgeon to the patient [19]. In fact, it has been shown that a well-informed patient tends to have more realistic expectations, heightened satisfaction, and even exhibits improved compliance with the prescribed treatment regimen [24]. Moreover, patient belief of being given sufficient information to make an informed choice is an essential component of the informed consent process. Improving the information transfer and patient’s knowledge of the surgical technique and its alternatives may even reduce malpractice claims. In fact, the majority of medical malpractice claims are linked to failures in communication and information transfer as opposed to failures of treatment [24, 25]. So that, we believe that the use of this video tool has relevant clinical implications in daily practice.

Unlike in previous studies conducted on other population samples [26, 27], no significant difference between the two groups was demonstrated in our study regarding anxiety although higher scores (indicating greater anxiety and higher decisional conflict) were recorded in the control group compared to the multimedial one. In this regard, a recent systematic review on the effectiveness of multimedia technology in enhancing outcomes (e.g., anxiety) in the preoperative education of adult cancer patients reported reduced anxiety, even though there was no noteworthy difference when compared to conventional methods. The decreased anxiety between groups, even though not significant, may suggest the use of multimedia content would be a good strategy to improve patients’ well-being and understanding, but combined with other impacting elements, such as physicians’ attitude and interpersonal characteristics able to improve the relationship and the confidence between healthcare providers and patients [28].

Similarly, the patients in our study reported low levels of decisional conflict, with no significant differences observed between the groups. Based on these results, we can speculate that the choice of breast reconstruction procedure may not be heavily influenced by how information is presented to patients. Existing literature indicates that patients often experience high levels of decisional conflict prior to consultations with physicians, especially when they have not yet had the chance to fully understand the harms and benefits of the surgical procedure [29, 30].In contrast, once patients receive the necessary information, their decision to undergo breast reconstruction surgery tends to be accompanied by less confusion. In this context, we can suggest that other personal (e.g., health literacy, age), psychosocial, and clinical factors play a more crucial role in facilitating patient understanding and decision-making process, regardless of the method used to convey that information, whether through printed materials or multimedia presentations [31, 32].

## Strengths and Limitations

Strengths of this study include the study design that created experimental groups with uniform characteristics, the large number of patients participating, the statistical analysis used to develop the project and supporting the results and the unique and innovative use of a multimedial tool for cancer patient candidates to mastectomy and implant-based reconstruction.

Moreover, another strength is that the plastic surgeon who visited the patient to plan breast reconstruction was blinded, he did not know which women were going to participate in the study and to which arm they were going to be allocated. Patients’ questionnaires were automatically collected by email preventing observer bias.

Finally, we tested the multimedial tool in non-hypothetical simulated scenarios but in a real-world study to evaluate its feasibility and full utility in daily practice.

Limitations include the use of a “pilot” questionnaire not validated in breast cancer patients. We developed this tool since no validated questionnaire is currently available in our native language about post-mastectomy reconstruction with implants. We tested and modified the current questions included in the comprehension questionnaire on the basis of a preliminary pilot study in which we analyzed the answers of 60 patients who previously underwent implant-based reconstruction at our institute (piloting phase).

We also limited participation in this study to women having an email address and able to use technology. We believe this is a limitation to the widespread use of this educational tool among cancer patients. However, in our practice, we do not experience particular limitations in enrolling older patients since for most of them their relatives (sons or nephews) were available as support in downloading and administering the video-informed consent.

## Future Directions

Future research could explore the use of the STAY questionnaire both before and after the consultation with the Plastic Surgeon, with and without the video intervention, to evaluate the impact of each informed consent approach on patient anxiety levels. This aspect was not addressed in our current investigation.

Additionally, assessing patient satisfaction with the video tool could provide valuable insights. This can be achieved by incorporating specific questions into the evaluation process, allowing patients to rate their experience on a structured scale after viewing the video.

Moreover, our institutional Quality Service is actively developing a series of educational, procedure-specific videos covering various breast reconstruction techniques. The goal is to establish a comprehensive video library accessible to patients through our institutional website, further enhancing patient education and engagement.

## Conclusions

Based on our findings, the multimedia video-assisted informed consent process significantly improves patient knowledge and awareness regarding implant-based breast reconstruction. This method is demonstrably more effective in facilitating information comprehension and retention than traditional communication approaches and should be considered a standard practice for all patients undergoing mastectomy and breast reconstruction.

## Supplementary Information

Below is the link to the electronic supplementary material.

Supplementary file1 Original Italian Version with subtitles (MP4 151003 kb)
